# The Role of Throat Packs in Orthognathic Surgery—A Systematic Review and Meta-Analysis

**DOI:** 10.1155/tswj/9229475

**Published:** 2025-02-10

**Authors:** Mehul Saha, Anupam Singh, Kalyana Chakravarthy Pentapati, Srikanth Gadicherla, Chithra Aramanadka, Adarsh Kudva

**Affiliations:** ^1^Department of Oral and Maxillofacial Surgery, Manipal College of Dental Sciences, Manipal Academy of Higher Education, Manipal, Karnataka, India; ^2^Department of Public Health Dentistry, Manipal College of Dental Sciences, Manipal Academy of Higher Education, Manipal, Karnataka, India

**Keywords:** gastric contents, orthognathic surgery, postoperative nausea and vomiting, throat pack, throat pain

## Abstract

Orthognathic surgery entails a high risk of blood ingestion, which causes postoperative nausea and vomiting (PONV). Throat packs are placed to combat this problem. However, the efficacy of throat packs in reducing blood ingestion and PONV is debatable. We aimed to review the existing literature and pool the estimates of the quality of gastric contents, PONV, and throat pain associated with and without the use of throat packs among patients undergoing orthognathic surgery. Globally recognized databases (PubMed, Scopus, Embase, CINAHL, and Web of Science) were searched to identify relevant studies, and 2 randomized controlled trials comprising 84 participants were included. A qualitative analysis of the gastric contents showed that throat packs are not practical barriers against the ingestion of blood during orthognathic surgery. The meta-analysis revealed that placement of throat packs during orthognathic surgery did not reduce the incidence of PONV (*p* value = 1) and caused higher postoperative throat pain (*p* value = 0.02). Thus, the current review provides no evidence in favor of throat packs during orthognathic surgery. The role of throat packs in preventing blood ingestion is questionable due to a limited number of studies. They play no significant role in preventing PONV and increase postoperative throat pain.

## 1. Introduction

Orthognathic surgery is a procedure performed to reposition the maxilla or mandible to enhance occlusal stability, improve facial proportions and esthetics, increase the retrolingual airway dimension, and enhance temporomandibular joint (TMJ) functions [1, 2]. These surgical procedures are usually carried out via an intraoral approach which entails placement of intraoral vestibular incisions. It involves osteotomy cuts; movement of the jaw bones as per the predetermined surgical plan; and stabilization with intermaxillary fixation and rigid fixation, followed by closure. Concomitant surgeries such as rhinoplasty, septoplasty, inferior turbinate reduction bone grafting, malar augmentation, distraction osteogenesis, TMJ surgery, or neck liposuction may be required to enhance the surgical outcomes [1, 3]. Hence, there is a high risk of blood and other debris (bone chips, screws, wire bits, irrigation fluid, etc.) to slip and get lodged in the throat, aspirated, or ingested. Also, ingestion of blood during surgery is known to induce postoperative nausea and vomiting (PONV) [4–7]. PONV can be distressing and a significant cause of concern to the patient as well as the surgeon as it can induce electrolyte imbalance secondary to dehydration, bleeding, esophageal rupture, or aspiration of gastric contents. All these adverse events are uncomfortable and unpleasant, leading to overall patient dissatisfaction and substantial morbidity [8–10].

Hence, to overcome these problems and reduce the morbidity of the patients, surgeons often place throat packs. These packs are commonly inserted in the oropharynx or hypopharynx with the primary intention of absorbing blood and irrigation fluids and also act as a mechanical barrier to avoid the ingestion or aspiration of surgical debris and foreign bodies. Throat packs aim to minimize the incidence of PONV and other consequences [4–6, 11, 12].

There are documented limitations with the usage of throat packs, such as postoperative throat pain and dysphagia, injury to the mucosa and pharyngeal plexus, aphthous stomatitis, and tongue swelling, and may rarely result in fatal airway obstruction [5, 12–16]. These adverse effects can hinder postoperative oral intake, contributing to electrolyte imbalance and dehydration, thus outweighing the probable benefits of throat packs in the prevention of PONV [17].

Therefore, the use of throat packs has been controversial and a topic of debate. A few questionnaire studies have explicitly documented the magnitude of adverse effects associated with throat packs and the lack of consensus regarding their use, resulting in the overall opinion that throat packs should not be routinely used during nasal, sinus, upper airway, and otolaryngological surgeries [18–20]. However, throat packs are still widely used during orthognathic surgery.

A few recent systematic reviews (SRs) evaluated the need for throat packs in patients undergoing ENT, otolaryngologic, oral, and dental surgery. The authors could not elicit any clinical benefits of pharyngeal packs and advised against their use during ENT, otolaryngologic, and dental surgery [17, 21–23]. However, the heterogeneous population included in these reviews does not focus on the effectiveness of these packs among patients undergoing orthognathic surgical procedures, which are mainly performed intraorally with a high risk of blood and debris ingestion or aspiration. With this background, we aimed to review the existing literature and pool the estimates of the quality of gastric contents, PONV, and throat pain associated with and without throat packs among patients undergoing orthognathic surgical procedures.

## 2. Materials and Methods

### 2.1. Obtaining Eligible Studies

The SR and meta-analysis (MA) was reported as per the “PRISMA” 2020 guidelines [24]. The protocol for this review was registered with PROSPERO (CRD42024508844).

Globally recognized databases (“PubMed, Scopus, Embase, CINAHL, and Web of Science”) were electronically searched without date restriction, and potentially eligible studies pertaining to the effectiveness of throat packs, pharyngeal packs, or oropharyngeal packs in oral and dental surgery were included. A combination of free text words and keywords with the help of Boolean operators was used to search the databases—(“oral and maxillofacial surgery”, “throat pack”, “pharyngeal pack”, and “oropharyngeal pack”). The search strategy used for PubMed was ((“oral and maxillofacial surgery”[All Fields] AND ((“pharynx”[MeSH Terms] OR “pharynx”[All Fields] OR “throat”[All Fields]) AND pack[All Fields])) OR ((“pharynx”[MeSH Terms] OR “pharynx”[All Fields] OR “pharyngeal”[All Fields]) AND pack[All Fields])) OR ((“oropharynx”[MeSH Terms] OR “oropharynx”[All Fields] OR “oropharyngeal”[All Fields]) AND pack[All Fields]) (detailed search strategies for other databases is listed in Supporting Information 1).

### 2.2. Inclusion and Exclusion Criteria

The search was restricted to randomized controlled trials (RCTs) published or reported in English. Literature reviews, commentaries, brief communications, case reports, and case series were excluded. The inclusion criteria for the review were (A) RCT design studies, (B) patients undergoing orthognathic surgery under general anesthesia, and (C) studies comparing the sole intervention of application of a throat pack with nonapplication of a throat pack during orthognathic surgery under general anesthesia. The exclusion criteria were (A) literature reviews, case reports, and case series; (B) patients undergoing any other head and neck surgery; (C) studies comparing different types of throat packs or where throat pack application is not the sole intervention; and (D) nonavailability of full-length text in English. No restrictions were placed on outcome measures.

The search results were added to Rayyan, a web-based tool for screening titles and abstracts. This was performed by two reviewers independently. The included studies were subjected to full-text screening also by two reviewers independently. Discrepancies if any were resolved by a third review author.

### 2.3. Data Extraction and Synthesis

The information that was collected for data extraction included author names, year of publication, age, and sex distribution, type of anesthesia and surgery, type and location of the pack, postoperative quality of gastric contents, PONV, postoperative dysphagia (throat pain) and sore throat, and any other outcome measured. The eligible studies were subjected to quality assessment by two authors independently using the “Cochrane Risk of Bias” assessment tool [25].

### 2.4. Statistical Analysis

MA was performed using RevMan Ver.5.4.1 (Cochrane Collaboration, 2020). Heterogeneity was assessed using the *Q* and *I*^2^ statistics. Owing to the smaller number of studies, all the available study-level characteristics were re-examined and evaluated for consistency among these variables. Considering the lack of heterogeneity, the pooled estimates of dichotomous variables (PONV) were calculated using the Mantel–Haenszel method with a fixed-effect model and risk ratios were reported along with a 95% confidence interval. Pooled estimates of continuous variables (postoperative throat pain) were calculated using the inverse variance method with a fixed-effect model and mean difference with 95% confidence intervals was reported as recommended by Schulz et al. [26].

## 3. Results

**Figure 1:**
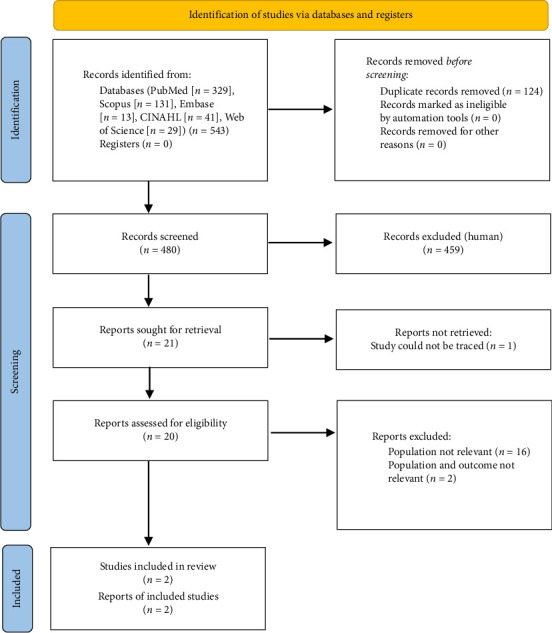
PRISMA flowchart showing the selection process of included studies.

A total of 543 publications were identified from various databases (PubMed [*n* = 329], Scopus [*n* = 131], Embase [*n* = 13], CINAHL [*n* = 41], and Web of Science [*n* = 29]). One hundred and twenty-four duplicates were removed and 480 articles were subjected to title and abstract screening. Twenty-one publications were eligible for full-text screening, of which one publication was unavailable after communication to the corresponding author [27]. Therefore, 20 publications were included in the full-text screening, and two publications [4, 5] were included for data extraction and risk of bias assessment based on the inclusion criteria (Figure 1).

A total of 84 participants were included in the SR–MA. The mean age reported by Faro et al. [4] was 29.44 (SD: 8.53) years (range: 18–48) with the majority being females (66%). The mean age reported by Powell et al. [5] was 29.17 (SD: 13.24) years (range: 16–64) with the majority being females (60%). In both studies, patients underwent orthognathic surgery under total intravenous general anesthesia with the placement of an oropharyngeal pack in the test group. The oropharyngeal pack consisted of four saline-soaked gauzes rolled without knots in the study by Faro et al. [4]. On the other hand, Powell et al. [5] used a sterile gauze secured with umbilical tape in their study without explicitly mentioning whether they were dry or soaked in saline or any other fluid. The outcomes measured by Faro et al. [4] were PONV (24 h), sore throat (2 and 24 h), and dysphagia (2 and 24 h), whereas Powell et al. [5] measured the quality of gastric contents before extubation, PONV (2 and 24 h), and throat pain (2 h) (Table 1).

### 3.1. Assessment of Risk of Bias

**Figure 2:**
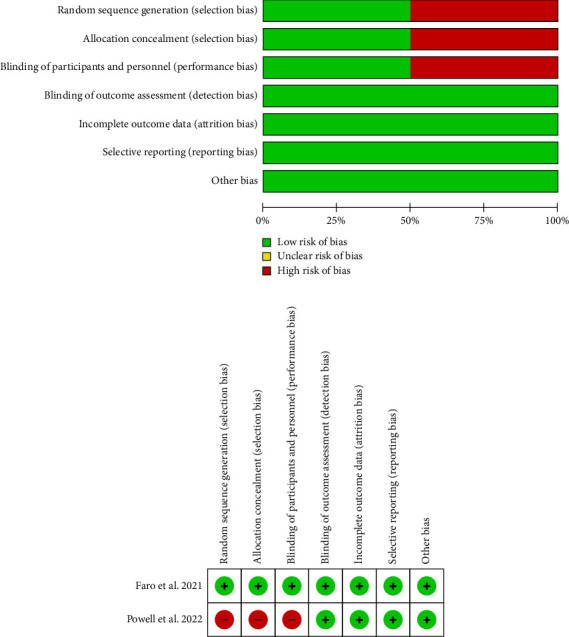
(a) Risk of bias assessment regarding each risk of bias item and summary of included studies. (b) Risk of bias assessment of individual studies.

Faro et al. [4] used a computer-generated program for randomization, and allocation concealment was performed with sequentially numbered opaque and sealed envelopes. Patients as well as the evaluator were blinded. Powell et al. [5] used odd and even birth years to randomize patients, and only patients were blinded to the assigned intervention, indicating a high risk of bias pertaining to randomization, allocation concealment, and blinding methods (Figures 2(a) and 2(b)).

### 3.2. Effect of Throat Pack on the Quality of Gastric Contents

Only Powell et al. [5] reported the quality of gastric contents. The operating surgeon aspirated gastric contents through a nasogastric tube (NGT) before extubation and classified it as bloody or not bloody. There was no significant difference between the two groups, and the gastric contents were bloody in a majority of the patients (66.7%) in both groups, thus favoring the conclusion that throat packs do not hinder the ingestion of blood or other fluids during orthognathic surgery (Table 2).

### 3.3. Effect of Throat Pack on PONV

Both the included studies evaluated PONV at different time points. Faro et al. [4] used the Kortilla scale to evaluate the incidence of PONV at 24 h whereas Powell et al. [5] evaluated self-reported PONV at 2 h and 24 h after surgery (Table 2).

**Figure 3:**
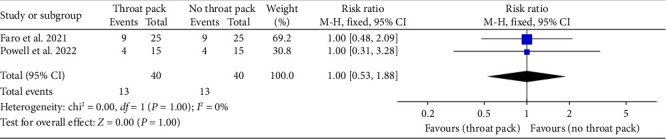
Meta-analysis and forest plot of incidence of PONV.

The MA showed that there was no significant difference in the incidence of PONV between the groups at 2 h (*p*-value = 1; RR = 1.00, 95% CI = 0.53–1.88; *I*^2^ = 0%) (Figure 3).

Thus, placement of throat packs during orthognathic surgery did not reduce the incidence of PONV. No analysis was performed for PONV at 24 h as only one study reported the finding. Further, Faro et al. [4] also evaluated the incidence of postoperative nausea (PON) and postoperative vomiting (POV) as separate parameters and found that while 34% of patients had PON, only 24% of patients had POV, indicating that nausea was not always followed by vomiting.

### 3.4. Effect of Throat Pack on Throat Pain

Both the included studies recorded throat pain at different time points. Faro et al. [4] recorded the incidence of throat pain at 2 and 24 h and Powell et al. [5] evaluated throat pain at 2 h. Both studies used the visual analogue scale (VAS) (Table 2).

**Figure 4:**
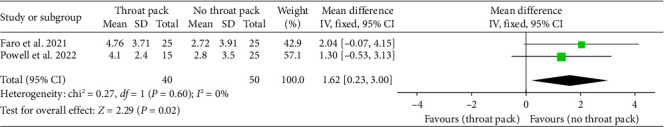
Meta-analysis and forest plot of incidence of postoperative throat pain.

The MA showed that the mean difference of pain was significantly higher among the group with throat pack than the group without throat pack after orthognathic surgery at 2 h with no heterogeneity among the studies (*p* value = 0.02; RR = 1.62, 95% CI = 0.23–3.00; *I*^2^ = 0%) (Figure 4). Thus, the placement of throat packs was shown to cause higher postoperative throat pain at 2 h. No analysis was performed for postoperative throat pain at 24 h as only one study reported the finding.

## 4. Discussion

This SR–MA evaluated the role of throat packs in the prevention of ingestion of blood and PONV during orthognathic surgery, and their effect on postoperative throat pain. Multiple trials reported the role of pharyngeal packs in PONV and postoperative throat pain in nasal, sinus, and upper airway surgery. These studies could not establish the efficacy of throat packs in reducing the frequency of PONV and showed a detrimental effect on throat pain [6, 11, 12, 28–32]. However, limited studies have evaluated the role of throat packs in minimizing the occurrence of PONV and their impact on throat pain in orthognathic surgery alone.

Overall, two studies with 84 participants were included in our MA. Faro et al. [4] evaluated PONV and throat pain with and without throat packs among patients undergoing orthognathic surgery. Powell et al. [5] investigated the possibility of ingestion of blood, PONV, and throat pain with and without throat packs in orthognathic surgery. The risk of bias was low for Faro et al. [4] and moderate to high for Powell et al. [5].

The usefulness of throat packs in preventing blood ingestion can be determined from the quality of aspirated gastric contents postoperatively. Only Powell et al. [5] studied gastric contents after orthognathic surgery to determine the role of throat packs in preventing the entry of blood into the stomach. They placed a 16-Fr NGT into the stomach and aspirated the gastric contents before extubation. The gastric contents were bloody in the majority of the cases (66.7%) in both groups, and no significant difference was seen between the two groups. However, the volume of gastric content aspirated was not reported. Postoperative gastric volume after nasal surgery was evaluated by Altun et al. [33], and they concluded that patients with intraoperative pharyngeal packs had lesser postoperative gastric volume (1.3 mL/kg) as compared to the control group without pharyngeal packs (1.95 mL/kg), and this in turn contributed to lesser PONV and sore throat in these patients. These findings were in agreement with Temel et al. [34] who studied the effect of pharyngeal packing on gastric volume and PONV in patients undergoing nasal surgery. They found that throat packs reduced the postoperative gastric volume and are useful in preventing intraoperative ingestion or aspiration of blood. However, neither of these studies evaluated the quality of the gastric contents, and therefore, no correlation exists between gastric volume and gastric contents.

Among the included studies, diverse methods were used to evaluate PONV. Faro et al. [4] used the Kortilla scale to measure PON, POV, and PONV at 24 h after surgery, while Powell et al. [5] evaluated self-reported PONV at 2 h and 24 h after surgery, and both these studies reported no significant difference in the prevalence of PONV with and without the use of throat packs. Our MA also indicated that there was no significant difference in the incidence of PONV between the groups at 2 h. These findings were in accordance with studies that evaluated the role of throat packs in preventing PONV among patients undergoing nasal, sinus, and upper airway surgery [6, 11, 12, 28–32]. Several SRs on the efficacy of throat packs among patients who underwent otolaryngological, nasal, and other head and neck surgery reported no beneficial effects in the reduction of PONV [7, 17, 21, 22].

PONV is multifactorial with multiple risk factors playing a significant role in its occurrence. The risk factors reported in the literature include female gender, younger age (< 50 years), nonsmoker, incidence of motion sickness or PONV in the past, type of anesthesia, type of surgery, duration of the procedure, intraoperative blood loss, and use of volatile anesthetics and postoperative opioids. The presence of NGT, anxiety, migraine, perioperative fasting, and body mass index (BMI) are some of the low-risk factors contributing to PONV [8–10, 35–37].

The type of surgery performed was similar in both studies, comprising LeFort 1 osteotomy for the maxilla, bilateral sagittal split osteotomy (BSSO) for the mandible, and genioplasty for the chin in various combinations. Silva, O'Ryan, and Poor [8] observed that the number of emetic episodes was the highest in bimaxillary (56.44%) followed by only maxillary (43.65%) and only mandibular (32.75%) orthognathic surgeries. Faro et al. [4] performed bimaxillary surgery with genioplasty (54%), bimaxillary surgery without genioplasty (40%), BSSO with genioplasty (2%), and only BSSO (4%) procedures. Powell et al. [5] performed bimaxillary surgery with genioplasty (13.3%), bimaxillary surgery without genioplasty (66.7%), only LeFort 1 surgery (16.7%), and only BSSO (3.3%) procedures. Thus, the distribution of type of surgery was similar in both the included studies.

Inhalational anesthesia with volatile anesthetics has the highest probability of inducing PONV [8, 10, 36]. However, in both the included studies, total intravenous general anesthesia was administered without the use of any volatile anesthetics. According to Gan et al. [35], one of the strategies to reduce PONV is the use of propofol to induce and maintain anesthesia, which was implemented in both the included studies. According to Silva et al. [8], longer procedures are associated with a higher occurrence of PONV with procedures lasting > 120 min having the highest prevalence of PONV (54.9%). In the study by Faro et al. [4], the average duration of surgery was 256.46 ± 74.55 min (range 204–600 min). Intraoperative blood loss may also play a role in PONV by disrupting the hemodynamic stability secondary to dehydration and electrolyte imbalance [4]. Bimaxillary surgery results in maximum blood loss due to the extensive vascularity of the maxillofacial region and long operative time [38]. Faro et al. [4] recorded an average blood loss of 588 ± 312.42 mL (range 100–1500 mL) and noted that 75% of the patients who had PONV had high blood loss volume of more than 500 mL.

The use of postoperative opioids is known to increase the occurrence of PONV [35, 36]. Faro et al. [4] advised intravenous morphine (0.1 mg/kg) as a postoperative analgesic whereas Powell et al. [5] advised short-acting opioids (fentanyl) or nonopioids (ketamine) for pain control. According to a study by Claxton et al. [39], patients who were advised morphine postdischarge had more incidence of PONV (59%) as compared to patients who were advised fentanyl (34%). Thus, the administration of postoperative morphine might have been a cause for higher PONV seen by Faro et al. [4]. In both studies, postoperative ondansetron was prescribed as the antiemetic to control PONV. Consequently, we may conclude that throat packs alone did not determine the presence or absence of PONV in these studies, as multiple other confounding factors affected this parameter. Also, there was significant bias as one of the included studies [5] had insufficient information regarding most variables.

Various studies that evaluated the effect of throat packs in nasal, sinus, and otolaryngological surgery reported the incidence of increased postoperative throat pain and dysphagia [6, 11, 12, 30–32, 40, 41]. However, Tay et al. [42] found no significant difference in throat pain when throat packs were placed or not placed during routine oral surgery. Arta et al. [43] also noted similar findings and recommended the use of pharyngeal packs during rhinoplasty. Our MA showed that the mean difference in pain was markedly higher among the group with throat packs than the group without throat packs after orthognathic surgery at 2 h, with no heterogeneity among the studies.

Several factors may contribute to throat pain with intraoperative throat packs. The packing material may itself cause injury to the mucosa, especially in longer surgeries, and result in throat pain [17]. Additional factors are the type and position of throat packs. Vural et al. [44] demonstrated that chlorhexidine gluconate 0.2% and benzydamine hydrochloride 0.15% (CGBH) soaked pharyngeal packs elicited less postoperative throat pain than saline-soaked pharyngeal packs during orthognathic surgery and a SR concluded the same [17]. On the contrary, Meco et al. [45] evaluated the efficacy of dry pharyngeal packs, water-soaked packs, CGBH-soaked packs, and no pharyngeal packs during sinonasal surgery. They found no significant difference in terms of postoperative sore throat. In our review, Faro et al. [4] used four saline-soaked gauzes and Powell et al. [5] used sterile gauze without explicitly mentioning whether they were dry or wet. However, in both studies, patients with throat packs had more throat pain than those without throat packs. Similar findings were reported in several other studies based on nasal and upper airway surgery [31, 40].

The position of the pack in the oropharynx, nasopharynx, or hypopharynx may also significantly affect postoperative dysphagia. Rizvi et al. [46] reported more dysphagia with oropharyngeal packing compared to nasopharyngeal packing. In both the studies included in our review, the authors have employed oropharyngeal packing, which could have contributed to higher pain scores. However, orthognathic surgeries are purely oral procedures with a high risk of blood and foreign body ingestion or aspiration, despite suctioning. Therefore, packing the oropharynx is justified in orthognathic surgeries. The position of throat packs can be a potential confounder which needs to be considered during the evaluation of throat pain secondary to throat packs. The position of throat packs may not be the same across all head and neck surgeries, due to which outcomes like gastric contents, PONV, and postoperative throat pain and discomfort may vary. Due to the above factors, it is not worthwhile to estimate the cumulative estimates of gastric contents, PONV, and postoperative pain and discomfort among patients undergoing all types of head and neck surgeries.

Several drawbacks of throat packs include mucosal injury, painful oral ulcers, injury to the pharyngeal plexus, edema of the tongue, pack retention, and in rare cases, fatal airway obstruction [12–16, 47]. These reports, along with the finding that throat packs do not reduce the occurrence of PONV, and in fact, lead to increased postoperative throat pain, have led to the consensus that throat packs should not be routinely used during sinus, nasal, and upper airway surgery, which has been corroborated by various SRs [7, 17, 21, 22, 48]. These SRs and meta-analyses have provided useful evidence against the placement of throat packs in all types of head and neck surgery [7, 17, 21, 22, 48] and have concluded that recent advances such as endoscopic techniques and hypotensive anesthesia have reduced the amount of intraoperative bleeding and hence circumvent the need for throat packs [21]. However, these reviews included a wide variety of surgical procedures. In addition, these reviews did not consider the quality of gastric contents. Few authors have recommended the application of throat packs when the risk of aspiration is high [40, 43]. Orthognathic surgery comprises a major bulk of oral and maxillofacial surgical procedures and entails a high risk of foreign body aspiration due to the use of mini-plates, screws, and wires of small dimensions and is hence very different from other head and neck surgeries.

Thus, in consideration of the above findings from the existing literature, we believe that future studies are required to evaluate the usefulness of throat packs in preventing foreign body aspiration, their effect on the volume and quality of gastric contents, their role in preventing PONV, and their influence on postoperative throat pain among patients undergoing orthognathic surgery. Also, trials need to consider and report findings related to the other potential risk factors of PONV, such as gender and age, type of surgery and anesthesia, position and type of throat pack, duration of the surgical procedure, intraoperative blood loss volume, and the use of postoperative opioids. This will help to lay down guidelines regarding the overall role of throat packs during orthognathic surgical procedures. The strength of the current review is the inclusion of patients chiefly undergoing orthognathic surgery and the inclusion of the quality of gastric contents as a determinant of throat pack effectiveness. A key limitation of the current review is the limited number of studies and the inclusion of studies published in the English language only.

## 5. Conclusion

In conclusion, the current review provides no evidence in favor of throat packs during orthognathic surgical procedures. The role of throat packs in preventing blood ingestion is questionable due to the limited number of studies. Throat packs play no significant role in preventing PONV, and they increase postoperative throat pain. We have, however, identified research gaps and believe that further studies are required to evaluate the true effectiveness of throat packs, especially pertaining to blood loss volume as a risk factor for PONV, the correlation between gastric volume and gastric contents to truly understand the barrier effect of throat packs and their role in the prevention of foreign body lodgment or aspiration during orthognathic surgical procedures.

## Figures and Tables

**Table 1:** Characteristics of included studies.

Authors and Year	Country	Type of study	Sample size	Surgery	Type of throat pack	Outcomes measured and time points	Conclusion
Faro et al. 2021 [4]	Brazil	Double-blind RCT	54	Orthognathic surgery: Bimaxillary surgery with genioplasty (54%) Bimaxillary surgery without genioplasty (40%) BSSO with genioplasty (2%) Only BSSO (4%)	Oropharynx: four saline-soaked gauzes rolled without a knot and connected with a cotton wire	PONV: 24 h Sore throat: 2 h & 24 h Dysphagia: 2 h & 24 h	Little to no benefit in decreasing PONV More sore throat and dysphagia in patients with throat packs
Powell et al. 2022 [5]	USA	Single-blind RCT	30	Orthognathic surgery: Bimaxillary surgery with genioplasty (13.3%) Bimaxillary surgery without genioplasty (66.7%) Only LeFort 1 surgery (16.7%) Only BSSO (3.3%)	Oropharynx: sterile X-ray-detectable gauze tied with umbilical tape	Quality of gastric content: prior to extubation PONV: 2 h & 24 h Throat pain: 2 h	Not effective barriers against the ingestion of blood Do not improve or worsen the incidence of PONV May increase throat pain

Abbreviations: BSSO, bilateral sagittal split osteotomy; h, hours; PONV, postoperative nausea and vomiting; RCT, randomized controlled trial.

**Table 2:** Data of included studies pertaining to quality of gastric contents, PONV, and throat pain.

Gastric contents	With throat pack		Without throat pack	
	Total (*n* = 15)		Total (*n* = 15)	
Powell et al. 2022 (prior to extubation)	Bloody	Not bloody	Bloody	Not bloody
	10	5	10	5

**PONV**	**Total (*n*)**	**Present**	**Total (*n*)**	**Absent**

Faro et al. 2021 (24 h)	25	9	25	9
Powell et al. 2022 (2 h)	15	3	15	3
Powell et al. 2022 (24 h)	15	4	15	4

**Throat pain**	**Total (*n*)**	**Mean** **±** **SD**	**Total (*n*)**	**Mean** **±** **SD**

Faro et al. 2021 (2 h)	25	4.74 ± 3.71	25	2.72 ± 3.91
Faro et al. 2021 (24 h)	25	3.52 ± 3.25	25	0.97 ± 2.27
Powell et al. 2022 (2 h)	15	4.1 ± 2.4	15	2.8 ± 3.5

Abbreviations: h, hours; PONV, postoperative nausea and vomiting.

## Data Availability

The authors confirm that the data supporting the findings of this study are available within the article and/or its Supporting Information.

## Supporting Information

Supporting Information 1: Detailed search strategies used for various databases: “PubMed, Scopus, Embase, CINAHL, and Web of Science”.

## References

[1] Robinson R. C., Holm R. L. (2010). Orthognathic Surgery for Patients with Maxillofacial Deformities. *AORN Journal*.

[2] Turnbull N. R., Battagel J. M. (2000). The Effects of Orthognathic Surgery on Pharyngeal Airway Dimensions and Quality of Sleep. *Journal of Orthodontics*.

[3] Khechoyan D. Y. (2013). Orthognathic Surgery: General Considerations. *Seminars in Plastic Surgery*.

[4] Faro T. F., de Oliveira E Silva E. D., Campos G. J., Duarte N. M., Caetano A. M. M., Laureano Filho J. R. (2021). Effects of Throat Packs during Orthognathic Surgery: a Double-Blind Randomized Controlled Clinical Trial. *International Journal of Oral and Maxillofacial Surgery*.

[5] Powell K., Amin D., Sesanto R., Bryant A., Kukreja P., Waite P. (2022). Do Oropharyngeal Throat Packs Prevent Fluid Ingestion during Orthognathic Surgery?. *International Journal of Oral and Maxillofacial Surgery*.

[6] Green R., Konuthula N., Sobrero M. (2017). Use of Pharyngeal Packs in Functional Endoscopic Sinus Surgery: A Randomized Controlled Trial. *The Laryngoscope*.

[7] Jin H. J., Kim S., Hwang S. H. (2019). Can Pharyngeal Packing Prevent Postoperative Nausea and Vomiting in Nasal Surgery?. *The Laryngoscope*.

[8] Silva A. C., O’Ryan F., Poor D. B. (2006). Postoperative Nausea and Vomiting (PONV) after Orthognathic Surgery: A Retrospective Study and Literature Review. *Journal of Oral and Maxillofacial Surgery*.

[9] Kovac A. L. (2000). Prevention and Treatment of Postoperative Nausea and Vomiting. *Drugs*.

[10] Apfel C. C., Eberhart L. J. H., Kranke P., Rüsch D. (2002). Recommendations for Randomized Controlled Trials to Prevent or Treat Postoperative Nausea and Vomiting. *Anästhesiologie & Intensivmedizin*.

[11] Yagueshita L., Lucinda L. R., Azevedo V., Minhoto Wiemes G. R., Minhoto Wiemes N. R., Polanski J. F. (2018). Audiologic Profile in Patients with Ankylosing Spondylitis: A Controlled Study of 30 Patients. *Ear, Nose & Throat Journal*.

[12] Basha S. I., McCoy E., Ullah R., Kinsella J. B. (2006). The Efficacy of Pharyngeal Packing during Routine Nasal Surgery: A Prospective Randomised Controlled Study. *Anaesthesia*.

[13] Mermer R. W., Zwillenberg D., Maron A., Brill C. B. (1990). Unilateral Pharyngeal Plexus Injury Following Use of an Oropharyngeal Pack during Third-Molar Surgery. *Journal of Oral and Maxillofacial Surgery*.

[14] Sharma P. K., Bhakta P., Srinivasan S., Khan R. M., Kaul N. (2012). Acute Tongue Enlargement Secondary to Pharyngeal Packing after Tracheal Intubation-A Case Report. *Middle East Journal of Anesthesiology*.

[15] Baranger V., Bon Mardion N., Dureuil B., Compère V. (2016). Human Error in Throat Pack Management. *A Case Reports*.

[16] Basha M. S. (2018). Missing Pharyngeal Pack Endoscopically Retrieved: An Avoidable Complication. *Annals of Maxillofacial Surgery*.

[17] Xie X., Yao Y. (2024). The Pharyngeal Packs for Dental and Otolaryngological Surgery: A Meta-Analysis of High-Quality Randomized Controlled Trials. *Ear, Nose & Throat Journal*.

[18] Piromchai P., Kasemsiri P., Reechaipichitkul W. (2020). Squeeze Bottle versus Syringe Nasal Saline Irrigation for Persistent Allergic Rhinitis: A Randomized Controlled Trial. *Rhinology*.

[19] Gupta A., Sarma R., Gupta N., Kumar R. (2021). Current Practices and Beliefs Regarding the Use of Oropharyngeal Throat Pack in India: A Nationwide Survey. *Indian Journal of Anaesthesia*.

[20] Bisase B., Matthews N. S., Lan C. (2011). Current Practice and Opinions Regarding the Use of Oropharyngeal Throat Packs in the United Kingdom. *Journal of Patient Safety*.

[21] Anderson C. R., Premakumar Y., Navaratnam A. V., Rouhani M., Singh A. (2020). The Use of Throat Packs in Ear, Nose and Throat, Oral and Dental Surgery: A Systematic Review. *Rhinology Journal*.

[22] Casenave T., Raynaud N., Geoffroy F., Torres J. H. (2023). Interest of Pharyngeal Packing in Head and Neck Surgery: A Meta-Analysis. *Journal of Oral Medicine and Oral Surgery*.

[23] Oppermann L. P., Lubianca Neto J. F., Drummond R. L. (2020). Hypopharyngeal Packing during Adenotonsillectomy by Cold Dissection in Children: a Randomized Controlled Trial. *European Archives of Oto-Rhino-Laryngology*.

[24] Page M. J., McKenzie J. E., Bossuyt P. M. (2021). The PRISMA 2020 Statement: An Updated Guideline for Reporting Systematic Reviews. *BMJ*.

[25] Higgins J. P. T., Altman D. G., Gøtzsche P. C. (2011). The Cochrane Collaboration’s Tool for Assessing Risk of Bias in Randomised Trials. *BMJ*.

[26] Schulz A., Schürmann C., Skipka G., Bender R. (2022). Performing Meta-Analyses with Very Few Studies. *Methods in Molecular Biology*.

[27] Tareen M. K., Hussain K., Anwar F. (2007). Postoperative Sore Throat after Oral Surgery under Endotrachial Intubations; Influence of the Pharyngeal Pack. *Medical Forum*.

[28] Piltcher O., Lavinsky M., Lavinsky J., de Oliveira Basso P. R. (2007). Effectiveness of Hypopharyngeal Packing during Nasal and Sinus Surgery in the Prevention of PONV. *Otolaryngology: Head and Neck Surgery*.

[29] Korkut A. Y., Erkalp K., Erden V. (2010). Effect of Pharyngeal Packing during Nasal Surgery on Postoperative Nausea and Vomiting. *Otolaryngology: Head and Neck Surgery*.

[30] Karbasforushan A., Hemmatpoor B., Makhsosi B. R., Mahvar T., Golfam P., Khiabani B. (2014). The Effect of Pharyngeal Packing during Nasal Surgery on the Incidence of Post Operative Nausea, Vomiting, and Sore Throat. *Iran J Otorhinolaryngol*.

[31] Fennessy B. G., Mannion S., Kinsella J. B., O’Sullivan P. (2011). The Benefits of Hypopharyngeal Packing in Nasal Surgery: A Pilot Study. *Irish Journal of Medical Science*.

[32] Al-lami A., Amonoo-Kuofi K., Kulloo P., Lakhani R., Prakash N., Bhat N. (2017). A Study Evaluating the Effects of Throat Packs during Nasal Surgery: a Randomised Controlled Trial. *European Archives of Oto-Rhino-Laryngology*.

[33] Altun D., Özkan-Seyhan T., Canbaz M. (2024). The Effect of Pharyngeal Packing on Gastric Volume in Patients Undergoing Nasal Surgery: a Randomised, Controlled Trial. *Journal of Laryngology & Otology*.

[34] Temel M. E., Totoz T., Erkalp K., Temel G. S., Selcan A. (2019). A Randomized, Double-Blind Study of the Ultrasound Assessment of the Effect of Pharyngeal Packing on Perioperative Gastric Volume in Nasal Surgery. *BMC Anesthesiology*.

[35] Gan T. J., Diemunsch P., Habib A. S. (2014). Consensus Guidelines for the Management of Postoperative Nausea and Vomiting. *Anesthesia & Analgesia*.

[36] Cruthirds D., Sims P. J., Louis P. J. (2013). Review and Recommendations for the Prevention, Management, and Treatment of Postoperative and Postdischarge Nausea and Vomiting. *Oral Surgery, Oral Medicine, Oral Pathology and Oral Radiology*.

[37] Phillips C., Brookes C. D., Rich J., Arbon J., Turvey T. A. (2015). Postoperative Nausea and Vomiting Following Orthognathic Surgery. *International Journal of Oral and Maxillofacial Surgery*.

[38] Piñeiro-Aguilar A., Somoza-Martín M., Gandara-Rey J. M., García-García A. (2011). Blood Loss in Orthognathic Surgery: A Systematic Review. *Journal of Oral and Maxillofacial Surgery*.

[39] Claxton A. R., McGuire G., Chung F., Cruise C. (1997). Evaluation of Morphine versus Fentanyl for Postoperative Analgesia after Ambulatory Surgical Procedures. *Anesthesia & Analgesia*.

[40] Pabst A., Müller D., Thiem D. G. E. (2022). Effects of Throat Packs in Upper Airway Surgery under Intubation Anesthesia: a Randomized Controlled Trial. *Clinical Oral Investigations*.

[41] Borna R., McCleary S., Wang L. (2022). Effect of Throat Pack Placement on the Incidence of Sore Throat and Postoperative Nausea and Vomiting in Septorhinoplasty Patients: A Randomized Controlled Trial. *Aesthetic Surgery Journal*.

[42] Tay J. Y. Y., Tan W. K. S., Chen F. G., Koh K. F., Ho V. (2002). Postoperative Sore Throat after Routine Oral Surgery: Influence of the Presence of a Pharyngeal Pack. *British Journal of Oral and Maxillofacial Surgery*.

[43] Arta S. A., Ghavimi M. A., Rahbar M., Ali-Maddadi Y., Zarandi A. (2019). Effect of Pharyngeal Pack on Postoperative Nausea and Throat Pain in Patients Undergoing Rhinoplasty. *Pesqui Bras Odontopediatria Clin Integr*.

[44] Vural Ç, Yurttutan M. E., Sancak K. T., Tüzüner A. M. (2019). Effect of Chlorhexidine/Benzydamine Soaked Pharyngeal Packing on Throat Pain and Postoperative Nausea & Vomiting in Orthognathic Surgery. *Journal of Cranio-Maxillofacial Surgery*.

[45] Meco B. C., Ozcelik M., Yildirim Guclu C. (2016). Does Type of Pharyngeal Packing during Sinonasal Surgery Have an Effect on PONV and Throat Pain?. *Otolaryngology: Head and Neck Surgery*.

[46] Singh R., Rizvi M., Rasheed M., Sarkar A. (2015). Effects of Different Types of Pharyngeal Packing in Patients Undergoing Nasal Surgery: A Comparative Study. *Anesthesia: Essays and Researches*.

[47] Erkalp K., Korkut Y. A., Meric A. (2010). Pharyngeal Packing Is a Predisposing Factor for Postoperative Aphthous Stomatitis in Nasal Surgery. *Otolaryngology: Head and Neck Surgery*.

[48] Athanassoglou V., Patel A., McGuire B. (2018). Systematic Review of Benefits or Harms of Routine Anaesthetist‐inserted Throat Packs in Adults: Practice Recommendations for Inserting and Counting Throat Packs: An Evidence‐based Consensus Statement by the Difficult Airway Society (DAS), the British Association of Oral and Maxillofacial Surgery (BAOMS) and the British Association of Otorhinolaryngology, Head and Neck Surgery (ENT‐UK). *Anaesthesia*.

