# Supplementary material for: The Role of Throat Packs in Orthognathic Surgery—A Systematic Review and Meta-Analysis
Source: ScientificWorldJournal. 2025 Feb 10;2025:9229475. doi: 10.1155/tswj/9229475 (PMC11832261; doi:10.1155/tswj/9229475)
Supplement: Supporting Information — Additional supporting information can be found online in the Supporting Information section. [file 9229475.f1.docx]

**Search Terms:**

1. **Oral and maxillofacial surgery:**

- Maxillofacial Surgery
- Surgery, Maxillofacial
- Oral Surgery
- Exodontics

1. **Maxillofacial orthognathic surgery:**

- Orthognathic Surgical Procedure
- Procedure, Orthognathic Surgical
- Procedures, Orthognathic Surgical
- Surgical Procedure, Orthognathic
- Surgical Procedures, Orthognathic
- Jaw Surgery
- Jaw Surgeries
- Surgeries, Jaw
- Surgery, Jaw
- Maxillo-Mandibular Surgery
- Maxillo Mandibular Surgery
- Maxillo-Mandibular Surgeries
- Surgeries, Maxillo-Mandibular
- Surgery, Maxillo-Mandibular
- Maxillofacial Orthognathic Surgery
- Maxillofacial Orthognathic Surgeries
- Orthognathic Surgeries, Maxillofacial
- Orthognathic Surgery, Maxillofacial
- Surgeries, Maxillofacial Orthognathic
- Surgery, Maxillofacial Orthognathic

1. **Throat, pharynx, pharyngeal:**

- Pharynxs
- Throat
- Throats

1. **Oropharynx, oropharyngeal:**

- Oropharynxs

1. **Pack ??**

**Search Strategy:**

**Pubmed: (oral and maxillofacial surgery, throat pack, pharyngeal pack, oropharyngeal pack)**

(("oral and maxillofacial surgery"[All Fields] AND (("pharynx"[MeSH Terms] OR "pharynx"[All Fields] OR "throat"[All Fields]) AND pack[All Fields])) OR (("pharynx"[MeSH Terms] OR "pharynx"[All Fields] OR "pharyngeal"[All Fields]) AND pack[All Fields])) OR (("oropharynx"[MeSH Terms] OR "oropharynx"[All Fields] OR "oropharyngeal"[All Fields]) AND pack[All Fields])

**Scopus:**

( "Maxillofacial Surgery" OR "Surgery, Maxillofacial" OR "Oral Surgery" OR "Exodontics" ) AND ( "Throat pack" OR "Pharyngeal pack" OR "Oropharyngeal pack" ) AND ( LIMIT-TO ( LANGUAGE , "English" ) )

**Embase: (Oral and maxillofacial surgery, throat pack, pharyngeal pack, oropharyngeal pack)**

'oral and maxillofacial surgery' AND 'throat pack'/exp OR 'throat pack'

**Cinahl: (Oral and maxillofacial surgery, throat pack, pharyngeal pack, oropharyngeal pack)**

Oral and maxillofacial surgery, throat pack, pharyngeal pack, oropharyngeal pack

**Web of science: (Oral and maxillofacial surgery, throat pack, pharyngeal pack, oropharyngeal pack)**

ALL=(oral and maxillofacial surgery) AND ((ALL=(throat pack)) OR ALL=(pharyngeal pack)) OR ALL=(oropharyngeal pack)
